# Soluble PD-1 and PD-L1: predictive and prognostic significance in cancer

**DOI:** 10.18632/oncotarget.18311

**Published:** 2017-05-31

**Authors:** Xinxin Zhu, Jinghe Lang

**Affiliations:** ^1^ Department of Obstetrics and Gynecology, Peking Union Medical College Hospital, Peking Union Medical College, Chinese Academy of Medical Sciences, Beijing, China

**Keywords:** soluble, PD-1, PD-L1, tumor immunity, biomarker

## Abstract

The membrane-bound molecules programmed death 1 (PD-1) and its ligand PD-L1 (PD-1/PD-L1) belong to the immune checkpoint pathway. PD-1 pathway downregulates effector T cells in immune response, thereby causing immune suppression. Recent studies have revealed that membrane-bound PD-1 and PD-L1 also have soluble forms. These soluble forms increase the complexity and diversity of the composition and function of the PD-1/PD-L1 signaling pathway. However, the exact roles of these molecules remain unknown. The objective of this systematic review was to elucidate the biological significance of soluble PD-1/PD-L1 in human cancers and evaluate whether they are potential diagnostic, therapeutic, or prognostic biomarkers. We expect to provide new clues for future research on soluble PD-1/PD-L1 pathway in human malignant tumors.

## INTRODUCTION

Malignant tumor cells use various methods of immune suppression to resist antitumor immunity. One of these methods is modifying the PD-1/PD-L1 pathway, which is called the “immune checkpoint” [1]. The PD-1/PD-L1 pathway not only controls excessive immune activation normally, but also appears to be a means through which tumors evade the immune system [2]. The activation of this pathway can lead to tumor immune escape and promote tumor cell growth including the following aspects: T cell tolerance, T cell apoptosis, T cell exhaustion, enhancing immunosuppressive Treg cell function, inducible co-stimulatory molecule and PD-1 disbalance [3]. Therefore, immunotherapeutic agents that can improve the immune response or reduce immunosuppression are essential for treating human cancers. The current immunotherapies for malignant tumors include therapeutic vaccines, adoptive T cell transfer, immune modulators, cytokines, and immune checkpoint inhibitors [4]. The PD-1/PD-L1 immune checkpoint represents an available and promising pathway that can be blocked to reverse tumor-mediated immunosuppression [5–7].

PD-1 is mainly expressed on different immune cells and has two ligands, namely PD-L1 (B7-H1 or CD274) and PD-L2 (B7-DC or CD273) [8]. Although the interaction of PD-1/PD-L2 shows a 2–6-fold higher affinity compared with the interaction of PD-1/PD-L1, PD-L1 is regarded as the primary ligand of PD-1 [9]. Recently, the soluble forms of PD-1 and PD-L1 (sPD-1 and sPD-L1) have been detected in the blood of patients with tumors [10–12]. However, the roles of sPD-1 and sPD-L1 have not been fully elucidated. This systematic review discusses the production of sPD-1/PD-L1 and evaluates the physiological and pathological significance of their levels in human blood.

### Structure and production of mPD-1 and mPD-L1

T-cell immune responses are vital in adaptive antitumor immune responses through the direct killing of target tumor cells or indirect inhibition by cytokines [13]. The PD-1/PD-L1 pathway molecules including membrane-bound forms of PD-1/PD-L1 (mPD-1/PD-L1) and sPD-1/PD-L1 play immunosuppressive roles in tumor-driven T-cell immune responses [14–16]. To date, an increasing number of studies have demonstrated that the anti-mPD-1/PD-L1 monoclonal antibodies (mAbs) can reverse the immunosuppression and might serve as a promising clinical strategy for controlling human malignancies [17, 18]. We will first discuss the production and expression of the classic mPD-1/PD-L1 because the fundamental knowledge of mPD-1/PD-L1 facilitates the understanding of the PD-1/PD-L1 pathway and the roles of sPD-1/PD-L1.

### Structure and production of mPD-1

The *PD-1* gene was discovered in 1992 as an upregulated gene in a T-cell hybridoma and hematopoietic progenitor cell line [19]. The components of mPD-1, a 288-amino acid (aa, ∼55 kDa) type I transmembrane glycoprotein, include an immunoglobulin (Ig) superfamily domain, stalk of approximately 20 aa, transmembrane domain, and an intracellular domain of approximately 95 aa residues. The intracellular domain contains an immunoreceptor tyrosine-based switch motif (ITSM) and an immunoreceptor tyrosine-based inhibitory motif (ITIM). ITSMs are essential for the delivery of inhibitory signals [20]. The aa sequence of PD-1 near the C-terminal tyrosine is highly conserved between humans and mice and is related to the Src homology region 2 domain-containing phosphatase-1 (SHP-1) and SHP-2. On the contrary, the N-terminal tyrosine is not associated with either SHP-1 or SHP-2 [21].

PD-1 is expressed on active T cells, B cells, natural killer T cells, and myeloid cells such as dendritic cells (DCs), and activated monocytes [22, 23]. PD-1 expression can be induced following T-cell receptor (TCR)-mediated activation and stimulation by cytokines such as interleukin (IL)-2, IL-7, IL-15, and IL-21 [22, 24]. The mPD-1 glycoprotein is a coinhibitory molecule, which belongs to the CD28 family [25]. The expression of mPD-1 protein in a wide variety T lymphocytes is contrary to the limited expression of other CD28 family members, thereby implying that mPD-1 suppresses a wider range of immune responses than do other inhibitory members of the CD28 family [26]. The role of PD-1 in the promotion of immunity evasion and development and progression of several types of malignancies, including non–small-cell lung cancer (NSCLC) [27], melanomas [28], breast cancer [29], renal cell carcinomas (RCCs) [30], and Hodgkin disease [31]has been confirmed .

### Structure and production of mPD-L1

The two ligands of PD-1 are the B7 family molecules, PD-L1 (B7-H1, CD274) and PD-L2 (B7-DC, CD273) [9]. PD-L1 is a 290-aa type I transmembrane glycoprotein that consists of IgC- and IgV-like extracellular domains, signal sequence, transmembrane domain, and intracellular domains [22].

The expression profile of PD-L1 is considerably more promiscuous than that of PD-1. Cells of the hematopoietic lineage, including active T cells, B cells, NK cells, DCs, monocytes, and macrophages, can express mPD-L1. It is also expressed on activated vascular endothelial cells, cultured bone marrow–derived mast cells and mesenchymal stem cells [32]. Furthermore, in humans, mPD-L1 is extensively expressed in the tonsils, placental syncytiotrophoblasts, and lungs, where it mediates human immune tolerance [33] and human malignancies of the kidney [34], ovary [35], pancreas [36], stomach [37], esophagus [38], and liver [39]. PD-L1 mRNA or mPD-L1 protein can be upregulated by cytokines, such as IFN-γ, IL-4, IL-10, VEGF, hypoxia-inducible factor-1α, and the constitutive oncogene pathways, including IFN-γ/JAK2/IFN, PI3K, and MEK/ERK/STAT1 [40, 41]. The expression levels of mPD-L1 on tumor cells tend to be associated, to some extent, with cancer progression and are predictive biomarkers of tumorigenesis, unfavorable outcomes, and improved response to treatment with PD-1/PD-L1 blockades in gastric [42], breast [43], renal [44], and ovarian cancers [35].

### Mechanism of PD-1/PD-L1 signaling

**Figure 1:**
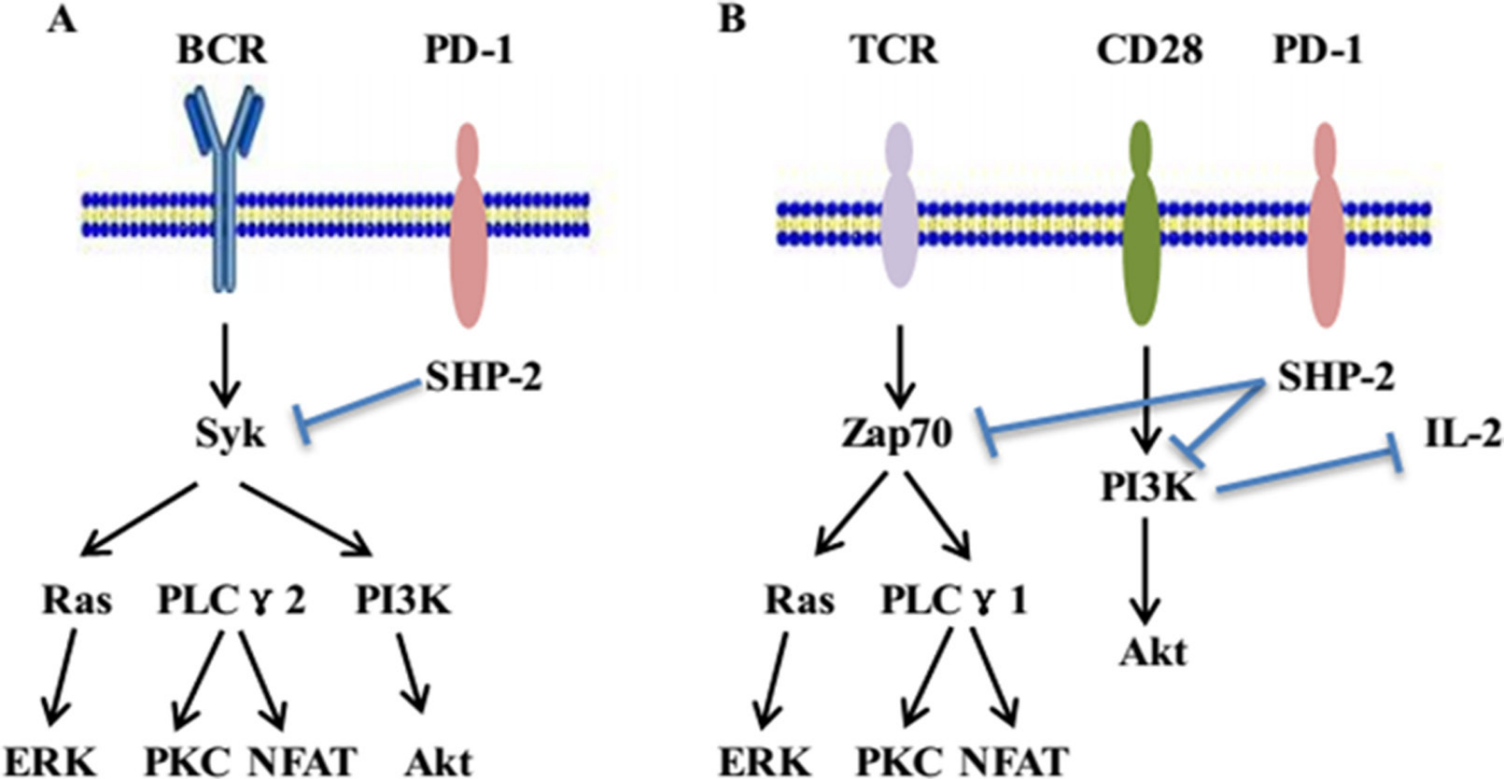
The inhibitory signaling of PD-1 pathway (**A**) In B cells, PD-1 ligation along with BCR signaling leads to the phosphorylation of the tyrosine residue in ITSM. The phosphorylated tyrosine residue in ITSM recruits SHP-2. Thereafter, SHP-2 dephosphorylates BCR-proximal signaling molecules, including Syk, which attenuate the activation of downstream molecules. (**B**) In T cells, PD-1 ligation along with TCR signaling results in the phosphorylation of the tyrosine residue in ITSM and recruits SHP-2. The recruitment of SHP-2 dephosphorylates signaling through the PI3K or Zap70 pathways and inhibits of downstream signaling. Meanwhile, blockade of PI3K activation and the subsequent downregulation of IL-2 further induces CD8+ and CD4^+^ T-cell anergy.

The inhibitory signaling of the PD-1 pathway was initially analyzed in the B cell line, IIA1.6, by using a chimeric molecule consisting of a PD-1 cytoplasmic region and an IgG Fc receptor type IIB extracellular region (FcPD). In the IIA1.6 cell line, co-crosslinking of B cell receptors (BCRs) and FcPD induced the phosphorylation of tyrosine residues in both ITSM and ITIM. Only the phosphorylated tyrosine residue in ITSM recruits SHP-2 through its SH2 domain and later phosphorylates the SHP-2 molecule. Subsequently, phosphorylated SHP-2 dephosphorylates the proximal well-known signal transducers of BCR, such as Ig α/β and Syk, which cause the deactivation of downstream molecules, including PLCγ2, PI3K, and ERK. Finally, the deactivated signal transducers lead to the suppression of acute-phase responses such as Ca^2+^ mobilization as well as long-term effects such as cell growth retardation [45]. In summary, these results indicate that PD-1 suppresses BCR signals by recruiting the SHP-2 molecule to its phosphotyrosine and dephosphorylating vital signal transducers of BCR signaling (Figure 1).

In T cells, the PD-1 pathway attenuates TCR signals through similar mechanisms. PD-1 downregulates the effector phase of T-cell immune responses by elevating the threshold of T-cell activation, inhibiting T-cell proliferation, and promoting activated T-cell apoptosis. The mechanism of PD-1 signaling is associated with dephosphorylation of molecules belonging to the TCR pathway and the transmission of suppressive signals [20]. PD-1 ligation, along with TCR signaling leads to the phosphorylation of the cytoplasmic region tyrosine, followed by the recruitment of SHP-2 to C-terminal tyrosine in the ITSM. Thereafter, SHP-2 dephosphorylates TCR-related zeta-chain-associated protein kinase 70 (ZAP70) and the CD28-related PI3K pathway, thereby causing inhibition of downstream signaling (Figure 1). Moreover, blockade or inhibition of PI3K signaling activation and the subsequent cell survival gene Bcl-XL reduce IL-2 production and glucose metabolism. The downregulation of IL-2 further induces the anergy in CD4^+^ T and CD8^+^ T cells [46, 47]. Notably, phosphorylated ITSM recruits SHP-1 in addition to SHP-2, whereas the contribution of SHP-1 to the suppressive function of PD-1 is considerably less than that of SHP-2[48].

### Production of sPD-1 and sPD-L1

Numerous costimulatory molecules in immunoregulation pathways assume two forms of expression, namely the membrane-bound and soluble forms. The soluble form of molecules is usually generated by proteolytic cleavage of the membrane-bound form of the costimulatory proteins, as in the case of soluble tumor necrosis factor [49] and sB7-H3 [50], or by translation of alternative spliced mRNA, as in the case of sB7–2 [51] and sCTLA-4 [52]. Similarly, the presence of soluble forms of PD-1/PD-L1 has been confirmed. Increasing evidence suggests that the blood levels of sPD-1/PD-L1 might facilitate the prediction of clinicopathological characteristics, treatment response, and survival outcomes in patients with cancer [12, 53, 54].

### Production of sPD-1

**Figure 2:**
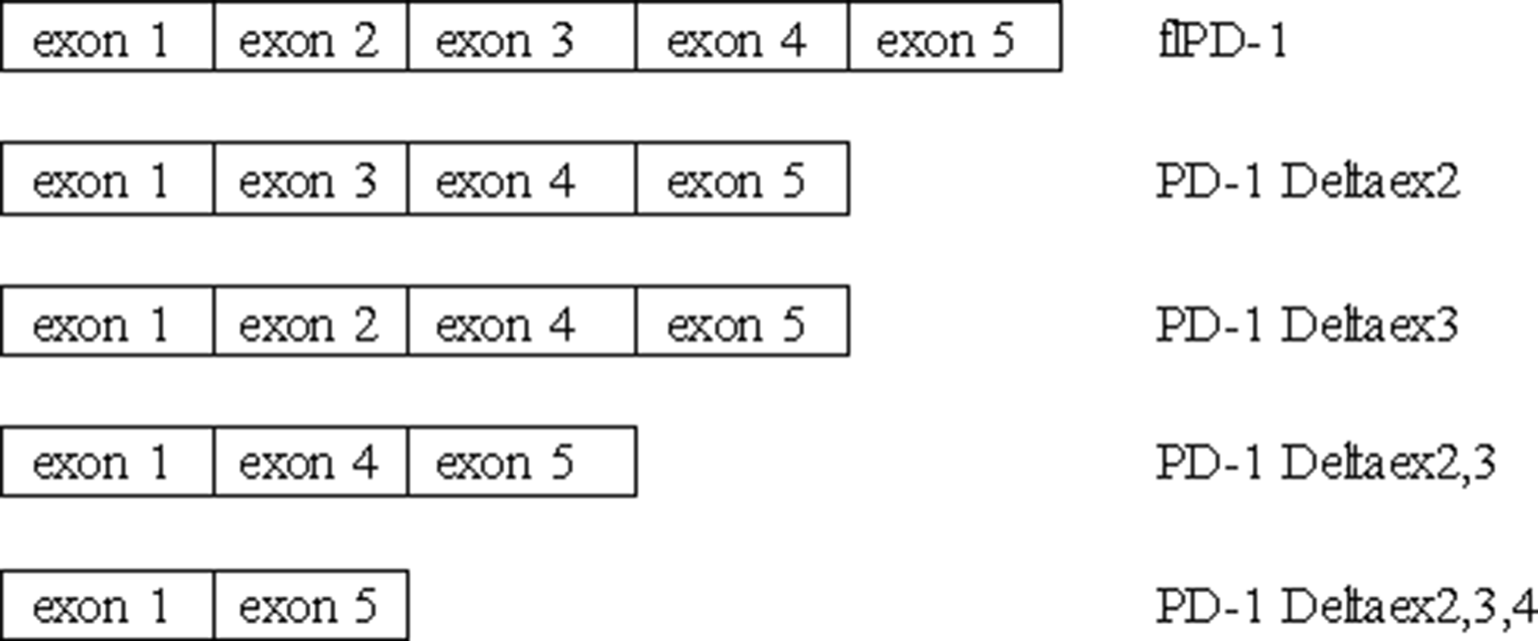
Different PD-1 splice variants Five splice variants of PD-1 mRNA transcripts have been cloned from human peripheral blood mononuclear cells: flPD-1, PD-1 Deltaex2, PD-1 Deltaex3, PD-1 Deltaex2,3, and PD-1 Deltaex2,3,4.

Five splice variants of PD-1 mRNA transcripts have been cloned from human peripheral blood mononuclear cells: flPD-1, PD-1 Deltaex2, PD-1 Deltaex3, PD-1 Deltaex2,3, and PD-1 Deltaex2,3,4 (Figure 2). The flPD-1 transcript demonstrated complete homology with the mPD-1 sequence. PD-1 Deltaex2 and PD-1 Deltaex3 are generated by alternative splicing where exon 2 and 3 (extracellular IgV-like domain and transmembrane domain, respectively) are spliced out. PD-1 Deltaex2,3 lacks exon 2 and 3. PD-1 Deltaex2,3,4 lacks exon 2, 3, and 4 (intracellular domain) and includes a premature stop codon in exon 5 [55]. PD-1 Deltaex2, PD-1 Deltaex2,3, and PD-1 Deltaex2,3,4 cannot bind to their ligands because of the lack of exon 2. The PD-1 Deltaex3 variant encodes the sPD-1, which is not detectable in healthy individuals [55, 56].

### Production of sPD-L1

The sPD-L1, is detectable in supernatants from mPD-L1^+^ cell lines rather than in those from mPD-L1^−^ cell lines [57], thereby indicating that mPD-L1 that is expressed on the cell surface might be a source of sPD-L1. However, other sources of sPD-L1 cannot be excluded [58]. Researchers have found that both immune [59] and tumor cells can be sources of sPD-L1 [10, 60, 61]. Among the immune cells, activated T cells and myeloid cells exhibit an increased level of mPD-L1, but the release of sPD-L1 is a feature of myeloid-derived cells [59]. This suggests that a regulatory mechanism that is different from that of mPD-L1 may control the production of sPD-L1. Moreover, no association was discovered between tumor PD-L1 expression (immunohistochemical analysis) and sPD-L1 levels (enzyme-linked immunosorbent assay analysis) in patients with diffuse large B-cell lymphomas (DLBCLs) and renal cell carcinomas, which indicated that the tumor microenvironment, including nonmalignant cells, may also generate sPD-L1 [62, 63].

Furthermore, sPD-L1 was expressed in normal human serum and the levels of sPD-L1 in human serum increased with age. Children aged 3–10 years had the lowest expression levels of sPD-L1, whereas adults aged 51–70 years had the highest levels (0.725 ± 0.181 ng/mL vs 1.040 ± 0.681 ng/mL, respectively) [57]. Moreover, the sPD-L1 levels might increase when matrix metalloproteinases (MMPs) cleave the extracellular fraction of mPD-L1; therefore, the production of sPD-L1 is suppressed by MMP inhibitors [20, 57]. However, whether the cleavage occurs randomly or is controlled by unique mechanisms remains to be determined.

### Functions of sPD-1 and sPD-L1

Tumor-related mPD-1 and mPD-L1 contribute to tumor immune escape [64, 65]. Immunotherapeutic strategies against malignant tumors using their antagonists have aroused researchers’ intense interest. The sPD-1/PD-L1 may represent unanticipated elements that contribute to immune response as well.

### Functions of sPD-1

Several studies on the functions of sPD-1 are based on animal models. In a study on murine hepatocarcinoma, the eukaryotic expression plasmid encoded sPD-1 protein was constructed, and its significant improvement in the antitumor immunity was proven [66]. Later, researchers used the adeno-associated virus to deliver sPD-1 into tumor sites, which inhibited H22 hepatoma cell growth, enhanced lysis of cancer cells and finally prolonged the overall survival (OS) of tumor-bearing mice [67].

**Figure 3:**
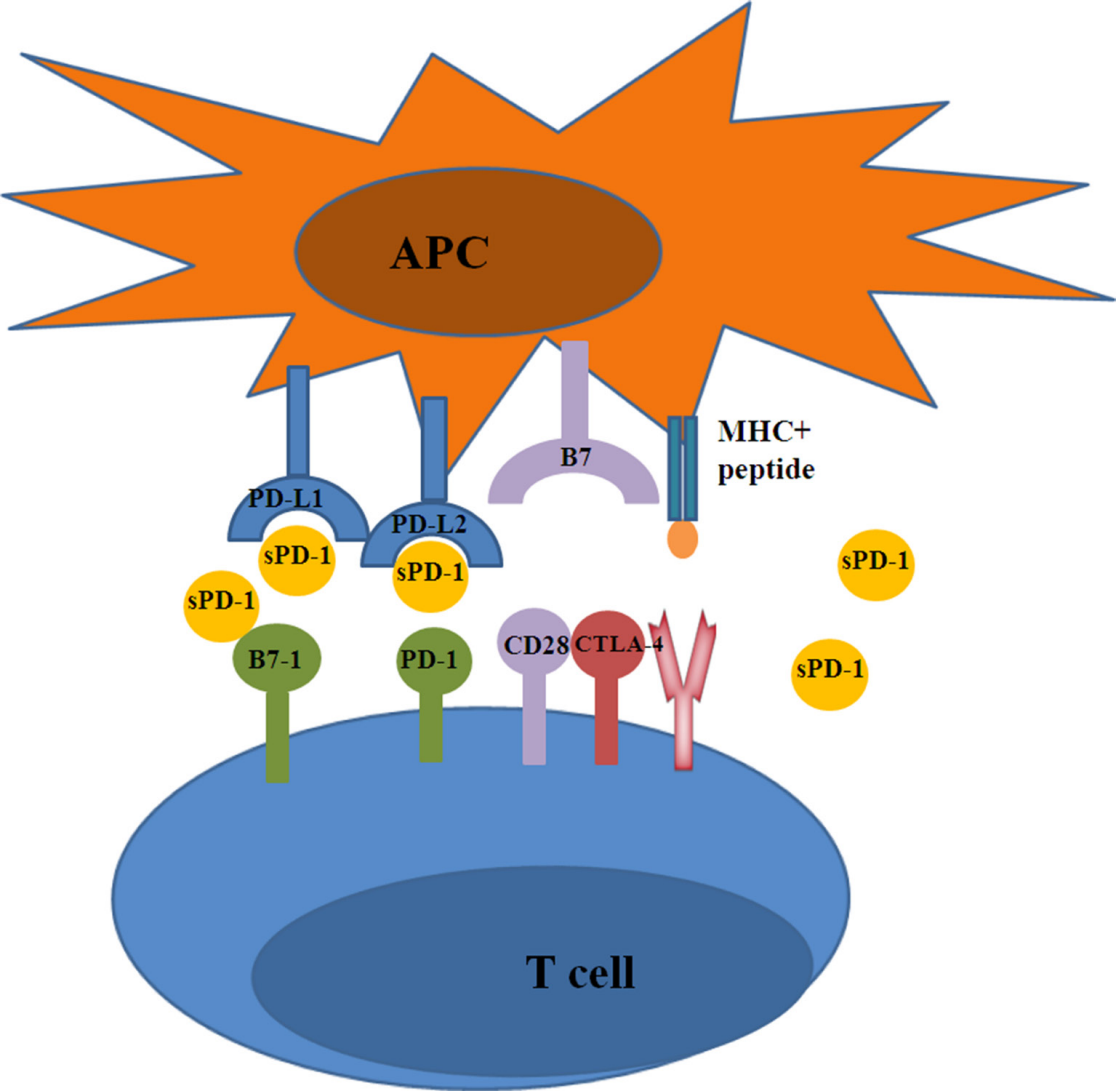
The blocking function of sPD-1 sPD-1 promotes T cell responses through blocking the following three interactions: PD-L1:B7-1, PD-L1:PD-1, and PD-L2:PD-1.

As aforementioned, the importance of the PD-1 checkpoint pathway in T-cell anergy and immunosuppression has been confirmed[68]. In this regard, antibody-based checkpoint blockades to reverse T-cell function in cancer immunotherapies have a promising future. Several PD-1/PD-L1 pathway blocking monoclonal antibodies (mAb), such as nivolumab [69], pembrolizumab [70], pidilizumab [71], durvalumab [72], and atezolizumab [73], have either entered into clinical trials or have been approved for clinical treatment and have shown potent therapeutic efficacies in multiple advanced-stage tumors. Studies have hypothesized that binding of sPD-1 to mPD-L1/mPD-L2 may prevent mPD-1 from combining with PD-L1 and PD-L2, thereby counteracting mPD-1-mediated inhibitory effects on T cells [57]. On one hand, the PD-1/mPD-L1 combination could provide direct inhibitory signal for T cells in the immune response. On the other hand, a great deal of sPD-L1 could afford distant influence to the activated T cells by the intervention of PD-1/mPD-L1 interactions. Moreover, sPD-1 may be more effective and potent than anti-PD-1 mAb for the following reasons: first, sPD-1 can suppress all three combinations (PD-L1:PD-1, PD-L2:PD-1, and PD-L1:B7-1(CD80)) that inhibit T-cell immune responses (Figure 3) [74]. Second, unlike mAb immunotherapy, the sustained but lower level of serum sPD-1 may reduce side effects while still exerting a relatively strong therapeutic effect [75]. Third, sPD-1 is more cost effective than mAb as the latter necessitates high and frequent doses [74].

In addition, the use of sPD-1 combined with other agents produced promising outcomes *in vitro* experiments. Blockade of tumor PD-1 pathway by hydrodynamic injection of plasmid DNA encoding sPD-1 and tumor-derived heat shock protein-70 (HSP70) peptide complexes reduced pulmonary metastasis of B16F1 melanoma cells and therefore increased the OS rate in female C57BL/6 mice [75]. Similarly, the *in vitro* coadministration of human papilloma virus-16 E7 DNA vaccine with sPD-1 DNA considerably improved E7-specific CD8^+^ T-cell responses, leading to valid antitumor effects against E7-expressing tumors [74]. The codelivery of sPD-1 DNA increased the maturation of DCs, which was accompanied by upregulation of DC maturation markers such as major histocompatibility complex II (MHC II) [74]. Considering that DC maturation is mediated by activated T lymphocytes, we speculate that DC maturation upregulated by sPD-1 might be influenced by the augmentation of T-cell responses. All these findings indicate that the sPD-1 plays an adjuvant role in enhancing antigen-specific T-cell immunity responses.

A few clinical studies have analyzed the relationship between the level of sPD-1 in blood and clinicopathological characteristics in patients with cancer for evaluating the predictive role of sPD-1 (Table 1) [53, 76, 77]. In patients with NSCLC, a study investigated the change in concentration of sPD-1 in the blood at initiation of erlotinib treatment and at the time of clinical resistance to erlotinib. It reported that patients with increased sPD-1 levels during erlotinib treatment were associated with prolonged progression-free survival (*p* = 0.013) and OS (*p* = 0.006) [53]. By contrast, in hepatitis B virus (HBV)-related hepatocellular carcinoma (HCC), Cheng et al. [76] assessed the effect of sPD-1 levels on the long-term dynamics of HBV load and HCC risk. They found that the longitudinal effects of increased sPD-1 level maintained higher viral loads for 4 years or more, and plasma quartile sPD-1 levels were positively related to HCC risk in men. Additional studies are needed to explore the potential and precise mechanisms underlying the discovered association between sPD-1 and malignant tumors.

**Table 1:** Studies on clinical significance of sPD-1 and sPD-L1 in human cancer

	Tumor	n	Aim	Outcomes	Ref
**sPD-1**	NSCLC	38	To compare the concentration of sPD-1 in patients with EGFR mutation prior to erlotinib treatment and at the time of progression and to correlate these results to patient outcome.	1. The serum concentration of sPD-1 increased during erlotinib treatment; 2. An increase in sPD-1 during treatment was associated with prolonged PFS (adjusted HR = 0.32, *p* = 0.013) and OS (adjusted HR = 0.33, *p* = 0.006).	[53]
	OSCC	107	To detect the expression levels of sPD-1 in OSCC patients and to discuss their biological and clinical significance.	There was no difference between the OSCC group and the control group (*p* > 0.05).	[77]
	HCC	2903	To assess the impact of sPD-1 levels on long-term dynamics of HBV load and HCC risk.	1. The levels of sPD-1 were positively associated with HCC risk for men; 2. The elevated sPD-1 levels maintained higher viral load for 4 or more years; 3. High levels of viral load and sPD-1 was associated with a 6.29-fold increase in risk of HCC.	[76]
**sPD-L1**	OSCC	107	To detect the expression levels of sPD-L1 in OSCC patients and to discuss their biological and clinical significance.	1. The average of sPD-L1 was remarkably higher in OSCC group (*p* < 0.05); 2. sPD-L1 expression was related to clinical stage, tumor cell differentiation, and lymph node status (*p* < 0.05).	[77]
	NNKTL	40	To examine the expression of sPD-1 in NNKTL.	1. The sPD-L1 level was significantly increased in NNKTL patients (*p* = 0.0074); 2. The high-sPD-L1 group of patients showed significantly worse 5-year OS than the low-sPD-L1 group (*p* = 0.0332).	[12]
	MM	96	To serum sPD-L1 levels in patients.	1. MM patients had higher sPD-L1 concentrations than healthy controls (*p* < 0.0001); 2. The overall response rate to treatment was higher in low sPD-L1 patients than in high sPD-L1 patients (*p* = 0.006); 3. Patients with lower sPD-L1 levels had higher 3-year PFS and OS rates (*p* < 0.05).	[54]
	NSCLC	174	To evaluate the association between sPD-L1 expression and clinical characteristics in patients with advanced NSCLC.	1. The expression of sPD-L1 in advanced NSCLC patients was significantly upregulated compared with the healthy control (*p* < 0.001); 2. The expression of sPD-L1 was significantly correlated with abdominal organ metastasis (*p* = 0.004); 3. A high sPD-L1 expression had a worse prognosis than a low expression in patients (*p* < 0.001).	[3]
	ccRCC	172	To determine whether sB7-H1 levels in patients with ccRCC are associated with pathologic features and patient outcome.	1. Higher preoperative sB7-H1 levels were associated with larger tumors (*p* < 0.001), tumors of advanced stage (*p* = 0.017) and grade (*p* = 0.044), and tumors with necrosis (*p* = 0.003); 2. A doubling of sB7-H1 levels was associated with a 41% increased risk of death (*p* = 0.010).	[60]
	DLBCL	348	To evaluate the clinical impact of sPD-L1 level measured at the time of diagnosis for newly diagnosed DLBCL.	1. Patients with elevated sPD-L1 experienced a poorer prognosis with a 3-year OS (*p* < 0.001); 2. sPD-L1 was found elevated in patients at diagnosis compared with healthy subjects and its level dropped back to normal value after CR.	[62]
	NSCLC	588	To investigate the PD-L1 polymorphism and the level of sPD-L1 in NSCLC.	1. NSCLC patients showed increased levels of sPD-L1 compared to controls (*p* < 0.001); 2. Lung adenocarcinoma patients had higher sPD-L1 levels than patients with squamous cell carcinoma (*p* < 0.01); 3. No association was observed between the different genetic variants and plasma concentrations of sPD-L1.	[78]
	HCC	279	To investigate the prognostic value of sPD-L1 in HCC patients.	1. Soluble PD-L1 levels positively correlated with the stage of cirrhosis and with stages of HCC; 2.Patients with high serum sPD-L1 concentrations had an increased mortality risk (*p* < 0.001).	[79]
	MM	89	To analyze the levels of sPD-L1 in bone marrow plasma from 61 patients with MM at 100 days after AuHSCT.	The higher levels of sPD-L1 had a shorter response period (*p* = 0.002) as well as shorter OS (*p* = 0.043) than those in the normal-to-low-group.	[80]
	Lung cancer	96	To analyze the correlations of the sPD-L1 levels with clinico-pathological status, laboratory data, and survival of the patients.	1. OS was significantly reduced in patients with high compared with low sPD-L1 levels (*p* = 0.037); 2. Multivariate analysis revealed that high sPD-L1 levels were significantly related to poor prognosis (HR=1.99, *p* = 0.041).	[82]
	Gastric cancer	120	To evaluate the association between sPD-L1 expression and prognosis in patients.	1.The expression of sPD-L1 in cancer patients was significantly up-regulated compared with health people (*p* = 0.006); 2. The expression of sPD-L1 was significantly correlated with differentiation (*p* = 0.026) and lymph node metastasis (*p* = 0.041); 3. The adenocarcinoma patients with higher up-regulated PD-L1 expression had much better prognosis than low expression patients (*p* = 0.028).	[81]

AuHSCT: autologous hematopoietic stem cell transplantation; ccRCC: clear cell renal cell carcinoma; CR, complete remission; DLBCL: diffuse large B-cell lymphoma; EGFR: epidermal growth factor receptor; HBV: hepatitis B virus; HCC: hepatocellular carcinoma; HR: hazard ratio; MM: multiple myeloma; n: total number; NNKTL: nasal natural killer/T-cell lymphoma; NSCLC: non-small cell lung cancer; OS: overall survival; OSCC: oral squamous cell carcinoma; PFS: progression-free survival; Ref: reference; sPD-1: soluble programmed death 1; sPD-L1: soluble programmed death ligand 1.

### Functions of sPD-L1

In contrast to sPD-1, a higher number of studies has been conducted on the mechanisms and functions of sPD-L1 in humans than in animal models (Table 1) [3, 12, 54, 60, 62, 77–82]. At present, whether sPD-L1 can bind to PD-1, similarly to mPD-L1, and deliver an inhibitory signal is debatable. Wan et al. [56] showed that the levels of sPD-1 and sPD-L1 were considerably higher in the serum and synovial fluid samples of patients with rheumatoid arthritis compared with controls. *In vitro* experiments, CD4^+^ T lymphocytes were cocultured with synovial fluid mononuclear cells. The addition of PD-1 or PD-L1 fusion proteins at a concentration range higher than 20 ng/mL led to a significantly increased level of T-cell proliferation which can express mPD-1 or mPD-L1. This finding suggested that the properties of mPD-1 and mPD-L1 are upregulated by their soluble forms.

On the basis of the predictive role of sPD-L1, Zheng et al. [81] designed a study to evaluate the correlation between sPD-L1 level and the prognosis of patients with advanced gastric cancer. They discovered that the higher level of sPD-L1 was correlated with differentiated cancer and the absence of lymph node metastasis (*p* = 0.026 and *p* = 0.041, respectively). Moreover, in this study, the adenocarcinoma patients with higher levels of sPD-L1 had better prognosis than did low-level patients (*p* = 0.028). However, on the contrary, other studies have revealed that the sPD-L1 levels may indicate a poor prognosis or treatment resistance [62, 79, 82]. Frigola et al. [59] discovered that the exposure of CD4^+^ and CD8^+^ T lymphocytes to either tumor cell-derived sPD-L1 or mature DCs-derived sPD-L1 induced apoptosis. Similarly, Zhang et al. [3] reported that the average levels of sPD-L1 in patients with advanced NSCLC and healthy controls were significantly different (0.723±0.081 vs 0.565±0.048 ng/mL, respectively, *p* < 0.001). In addition, the median OS in the low- and high-level groups were 26.8 (95% CI: 26.2∼27.4 months) and 18.7 months (95% CI: 15.9∼21.5 months), respectively. This suggested that patients with a low sPD-L1 levels may have a longer survival time. A French multicenter clinical trial revealed that patients with elevated sPD-L1 experienced a poorer prognosis (*p* < 0.001) than did the controls, and the sPD-L1 level returned to its normal value after complete remission of DLBCL. Therefore, sPD-L1 may be a potent predicting biomarker in DLBCL, which indicates the utility of alternative therapeutic strategies [62]. Several other studies have also confirmed that sPD-L1 is a negative therapeutic and prognostic biomarker in malignant tumors, such as multiple myeloma [54] and renal cell carcinoma [60]. However, to date, the reasons of sPD-L1for the dichotomous effects on T lymphocytes remain unknown.

## CONCLUSIONS

In recent years, with the growth of economies and improvement in living standards, cancer incidence and mortality have increase significantly [83]. Cancer is the leading cause of death in China and is a major public health problem [84]. The detection and evaluation of cancer through mass screening and by using less invasive and more efficient markers are major public health requirements. Mass screening biomarkers, such as alpha-fetoprotein [85], antigen [86], carbohydrate antigen (CA) 19–9 and CA125 [87], prostate-specific antigen [88], and lactate dehydrogenase [89], are generally used in clinical practice; however, they do not accurately or completely predict diagnosis, disease development, immunotherapeutic sensitivity, and prognosis. Accumulated data indicates that sPD-1 and sPD-L1, which can be easily detected in clinical practice, may play significant roles in tumor pathogenesis, immune responses, and prediction. Therefore, sPD-1/PD-L1 may provide new biomarkers and insight into potential treatment strategies including immunotherapy in malignant tumor.

In addition, the treatment strategies targeting PD-1/PD-L1 with mAbs such as nivolumab, pembrolizumab, and atezolizumab and so on exhibited promising results and fundamentally changed cancer treatments [90]. However, apart from the dichotomous effect of sPD-L1, the PD-1 pathway is complex and the molecular mechanisms remain unclear. Whether the PD-1 pathway mAbs can upregulate the antitumor responses of sPD-1/PD-L1 need further study. Moreover, the synergistic therapeutic effects of sPD-1 in combination with other antitumor agents, such as HSP70 peptide vaccine [75] and IL-21 [91], provide a new direction for future antitumor strategies.

The PD-1 pathway has generated immense academic and commercial interest in the fight against cancer [92]. The application of sPD-1 and sPD-L1 in the treatment of tumors is defined to date, but their initial results appear promising. We speculate that with the growing number of successful clinical trials associated with PD-1 pathway mAbs, additional investigations and studies on sPD-1 and sPD-L1 in cancers will soon be conducted.

### Ethical approval

This article does not contain any studies with human participants or animals performed by any of the authors.
